# Incidence and Predictors of Unexpected Malignancy in Benign Myomectomy or Hysterectomy

**DOI:** 10.7759/cureus.66880

**Published:** 2024-08-14

**Authors:** Fatema Y Sabt, Hasan M Isa, Zahra A Khudair, Enjy E Khedr, Fatema A Alkhan, Jumana S Hammad

**Affiliations:** 1 Department of Obstetrics and Gynecology, Salmaniya Medical Complex, Manama, BHR; 2 Department of Pediatrics, Arabian Gulf University, Manama, BHR; 3 Department of Pediatrics, Salmaniya Medical Complex, Manama, BHR; 4 Department of Public Health, Benha University, Benha, EGY; 5 Department of Obstetrics and Gynecology, Al Salam Specialist Hospital, Riffa, BHR

**Keywords:** uterine cervical neoplasm, ovarian neoplasm, endometrial neoplasm, sarcoma, uterine myomectomy, hysterectomy

## Abstract

Introduction

Detection of gynecological cancers preoperatively is imperative for practitioners for optimal patient management and outcome. This study aimed to estimate the incidence of unexpected malignancy (UM) in patients who underwent hysterectomy or myomectomy for presumed benign indications and to detect the predictive factors of UM.

Methods

A retrospective analytical study that included patients who underwent hysterectomy or myomectomy for benign indications from January 1st, 2016, to December 31st, 2020, was conducted at the Department of Obstetrics and Gynecology at Salmaniya Medical Complex, Bahrain. The main outcome was the overall incidence of UM and the incidence of each malignancy. Characteristics of UM were compared with benign pathologies. Fisher’s exact and Pearson's chi-square tests were used to compare categorical variables and the Mann-Whitney U test or student's t-test for continuous variables. Binary logistic regression was used to identify the predictors of occurrence of UM. Confidence interval (CI) was set at 95%. A probability value (p-value) less than 0.05 was considered statistically significant.

Results

Out of 513 patients who underwent hysterectomy or myomectomy, 379 (73.9%) fulfilled the inclusion criteria, 314 (82.8%) hysterectomies and 65 (17.2%) myomectomies. The overall incidence of UM was 1.3% (n=5/379), 1.3% (n=4/314) among hysterectomies and 1.5% (n=1/65) among myomectomies. Three (0.8%) pre-malignant pathologies were identified: one (0.26%) smooth muscle tumor of unknown malignant potential, leiomyoma with bizarre nuclei, and mucinous borderline tumor of endocervical type of ovary each. The types of UM were sarcomas in three (0.26%) patients (two (0.5%) leiomyosarcoma and one (0.26%) endometrial stromal sarcoma) and endometrial adenocarcinoma and ovarian cancer in one (0.26%) patient each. No significant difference was found between the characteristics of UM and benign pathologies.

Conclusion

Although this study demonstrated a low incidence of UM among both hysterectomies and myomectomies, the age at the diagnosis of our patients with UM was as young as 34 years of age, and sarcomas were the most common type of UM. Disconcertingly, none of the studied independent variables had significantly predicted the occurrence of UM.

## Introduction

Detection of gynecological cancers preoperatively is imperative for practitioners for optimal patient management and outcome. Surgical planning of patients with suspected or confirmed malignancy differs from those with benign pathologies, and this may alter the route of surgery, operating team members with the need for involvement of gynecologic oncologists, or the anatomy that needs to be excised intraoperatively [1]. Furthermore, the technique of the surgery may disseminate the malignant cells, upstage the cancer, and jeopardize the patient’s prognosis like in the case of rupture of malignant ovarian cyst or morcellation of uterine leiomyosarcoma [1-6]. Unfortunately, despite the plentiful investigations that have increased the detection rate of most gynecological cancers preoperatively, there are no reliable diagnostic methods for some cancers like borderline or low-grade ovarian cancers and asymptomatic endometrial cancers. In addition, distinguishing leiomyosarcoma from benign leiomyoma preoperatively based on clinical presentation or radiological imaging remains difficult [2,3,6].

In the literature, the reported incidence of unexpected uterine malignancy is variable (0-3.17%) [2,7-9]. Most of the previously conducted research focused on uterine leiomyosarcoma which is less prevalent than endometrial cancers [10]. The incidence of other occult gynecological malignancies, ovarian and cervical cancers, was even less investigated, and the limited available data showed that the incidence of ovarian and cervical cancers is 0.19% and 0.6%, respectively [10]. The related data are even more limited in the Middle East region, and to the best of our knowledge, there are no previously published studies tackling occult gynecological malignancies in this region. To fill this gap of knowledge, this study was conducted to estimate the incidence of unexpected malignancy (UM) in hysterectomies and myomectomies performed for presumed benign indications in Bahrain and to find the clinical characteristics and factors that might predict the occurrence of UM among these patients. We also described the types of UM and the prognosis of patients with UM.

## Materials and methods

This is a retrospective analytical study conducted at the Department of Obstetrics and Gynecology at Salmaniya Medical Complex (SMC), Bahrain, from 1st January 2016 to 31st December 2020. SMC is a governmental hospital and is the largest tertiary hospital in the country with the highest volume of patients. This hospital receives referrals from all primary healthcare centers and other private hospitals in the country. All healthcare services are available, accessible, and affordable to patients. The Department of Obstetrics and Gynecology has a unit of gynecology oncology in addition to other general and specialized units. 

Electronic medical records of all patients who underwent abdominal hysterectomy with or without removal of adnexa, vaginal hysterectomy, laparoscopic-assisted vaginal hysterectomy, and abdominal, laparoscopic, or vaginal myomectomy were reviewed by a gynecologist. All cases that were performed for benign indications were included in the study. These indications included abnormal uterine bleeding, adnexa mass or ovarian cyst, leiomyoma, adenomyosis, endometriosis, and pelvic organ prolapse. UM was defined as a malignant condition diagnosed on postoperative pathological specimens without preoperative diagnosis or clinical suspicion of malignancy. Patients who underwent surgery for obstetric indications, for suspected or biopsy-confirmed malignancy or atypia, or those in whom the surgical team involved a gynecologic oncologist during the surgery were excluded. 

Demographic data of the included patients such as age, nationality, history of smoking, parity, menopause status, family history of cancer, and co-morbidities were collected. Indications of surgeries, preoperative workup including tumor markers, radiological imaging, histopathological reports of cervical cancer screen and endometrial biopsies, route of surgery (vaginal, laparoscopic, or abdominal), and the postoperative surgical pathology and their International Federation of Gynecology and Obstetrics (FIGO) stages were also gathered. For patients with unexpected pre-malignant or malignant pathologies, postoperative additional treatments such as the need for reoperation, radiotherapy, or chemotherapy, and the patients’ outcome (survival or death) along with their follow-up duration were reviewed. 

The collected data were entered into an Excel sheet and then transferred to Statistical Package for Social Sciences (SPSS) program version 21 (IBM Corp. (2012). IBM SPSS Statistics for Windows, Version 21.0. Armonk, NY: IBM Corp.) for analysis. Categorical variables were presented as frequency and percentages. According to their distribution normality, continuous variables were presented either as mean and standard deviation or median and interquartile range (IQR). The incidence of UM was calculated as the proportion of patients with UM over the total number of patients who underwent hysterectomies, myomectomies, and both. Moreover, the incidence of each type of UM (sarcoma, endometrial, ovarian, and cervical cancers) among hysterectomies and myomectomies was calculated. Patients with unexpected premalignant or malignant pathologies were compared with those with benign pathologies to detect factors that might be protective or predictive of UM. Categorical variables were compared using Fisher’s exact or Pearson's chi-square tests. Continuous variables were compared using the Mann-Whitney U test or Student's t-test. Binary logistic regression was used to identify predictors of the occurrence of UM. Confidence interval (CI) was set at 95%. A probability value (p-value) less than 0.05 was considered statistically significant.

This study conforms to the provisions of the Declaration of Helsinki in 1995 (revised in Edinburgh 2000) and was approved by the Research and Research Ethics Committee, SMC, Government Hospitals, Manama, Bahrain (Institutional Review Board number: 2100123).

## Results

**Figure 1:**
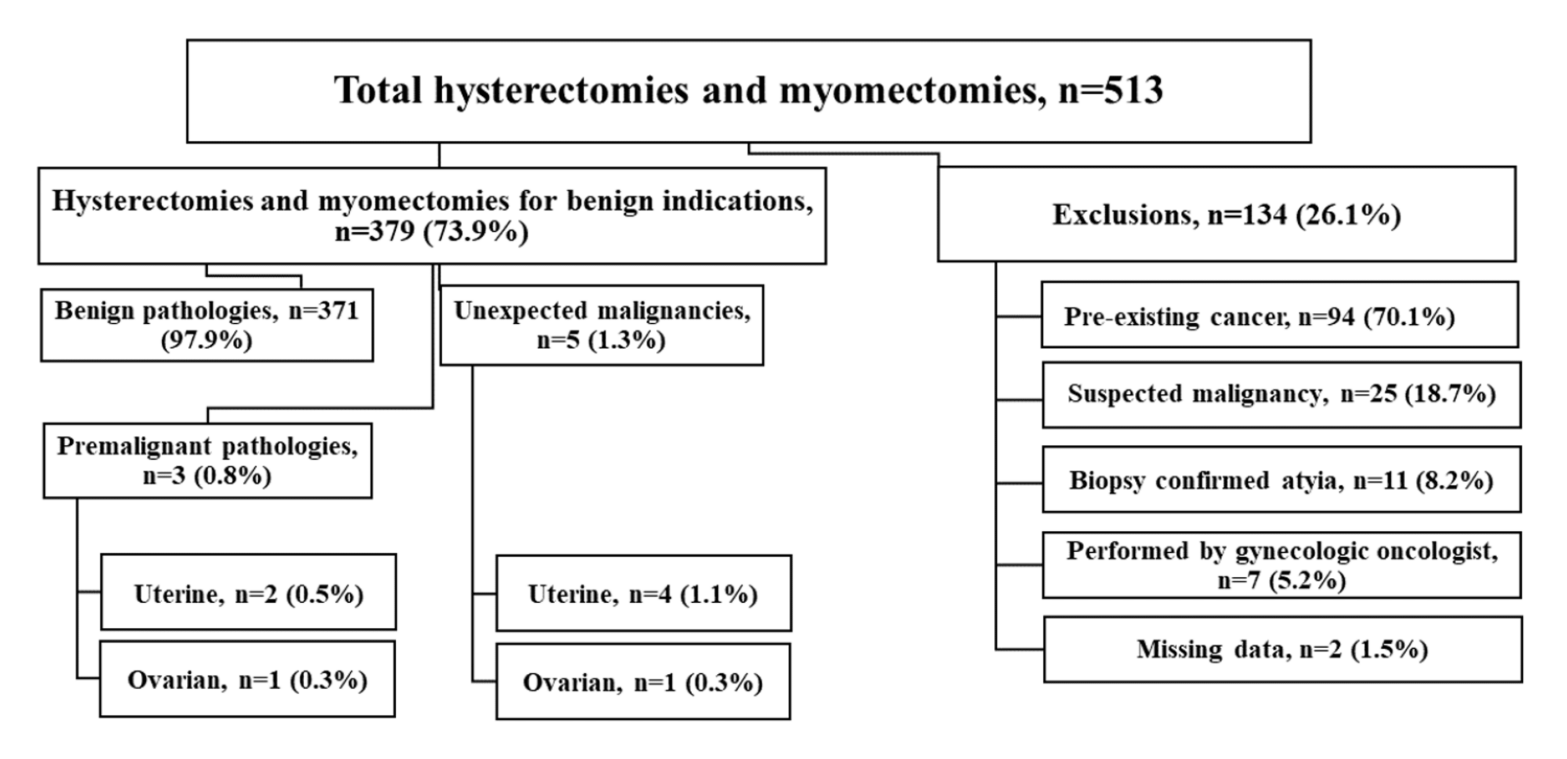
Flow chart of the study population The data has been represented as number (%). Image credits: Hasan M. Isa, Fatema Y. Sabt.

Out of 513 patients who underwent hysterectomy or myomectomy, 379 (73.9%) fulfilled the inclusion criteria, 314 (82.8%) hysterectomies and 65 (17.2%) myomectomies, and were included in the study (Figure 1).

The overall incidence of UM was 1.3% (n=5/379), 1.3% (n=4/314) among hysterectomies and 1.5% (n=1/65) among myomectomies. The median age at the time of surgery was 51 (IQR: 45-56) years (Table 1).

**Table 1:** Sociodemographic characteristics of 379 patients with benign versus patients with premalignant or malignant pathology The data has been represented as median (IQR) or number (%). ^a^Including a case of mucinous borderline tumor and a case of smooth muscle tumor of uncertain malignant potential. p-Values resulting from the ^b^Mann-Whitney test or the ^c^chi-square test and p-values <0.05 are considered statistically significant. UM, unexpected malignant and premalignant pathologies; yr, year; IQR, interquartile range; SCD, sickle cell disease; SD, standard deviation; BMI, body mass index.

Characteristics	Total, n =379	Benign, n =371	UM, n =8^a^	p-Value
	n (%)	n (%)	n (%)	
Age at procedure (yr), median (IQR)	51 (45-56)	50 (45-56)	48 (43-54)	0.501^b^
Nationality (n=379)				0.678^c^
Bahraini	298 (78.4)	292 (78.9)	6 (75.0)	
Non-Bahraini	80 (22.3)	78 (21.1)	2 (25.0)	
Smoking (n=11)	9 (82.0)	8 (80.0)	1 (50.0)	0.455^c^
Parity (n=333), median (IQR)	3 (1-5)	3 (1-5)	3 (1-4)	0.353^b^
None	62 (18.9)	60 (18.5)	2 (25.0)	0.766^c^
1-2	63 (18.9)	61 (18.8)	2 (25.0)	
≥3	207 (62.2)	203 (62.7)	4 (50.0)	
Menopause	156 (41.2)	153 (41.2)	3 (37.5)	1.000^c^
Abdominal pain (n=303)	86 (22.7)	82 (26.1)	4 (50.0)	0.23^c^
Abnormal uterine bleeding (n=301)	146 (38.5)	141 (44.9)	5 (62.5)	0.49^c^
Post-menopausal bleeding (n=233)	77 (20.3)	75 (23.9)	2 (25.0)	1.000^c^
Family history of malignancy (n=16)	6 (38.0)	4 (30.8)	2 (66.7)	0.518^c^
Comorbidity				
Diabetes	103 (28.0)	101 (28.1)	2 (25.0)	1.000^c^
Hypertension	118 (32.1)	116 (32.3)	2 (25.0)	1.000^c^
Dyslipidemia	75 (20.4)	73 (20.3)	2 (25.0)	0.669^c^
G6PD deficiency	44 (11.6)	43 (11.6)	1 (12.5)	1.000^c^
Hypothyroidism	49 (12.9)	48 (12.9)	1 (12.5)	1.000^c^
Asthma	16 (4.2)	16 (4.3)	0 (0)	1.000^c^
SCD	22 (5.8)	22 (5.9)	0 (0)	0.453^c^
BMI (n=198), median (IQR)	32.7 (32.2-27.2)	32.2 (27.1-36.1)	40.9 (27.4-41.2)	0.237^b^
Less than 18.5	2 (1.0)	2 (1.0)	0 (0.0)	0.936^c^
18.5-25	22 (11.1)	22 (11.3)	0 (0.0)	
25-29.9	60 (30.3)	59 (30.3)	1 (33.3)	
30 or more	114 (57.6)	112 (57.4)	2 (66.7)	

Most of the patients were Bahraini (n=296, 78.1%), while the remaining (n=83, 21.9%) were non-Bahraini (27 (7.1%) patients were from India, 25 (6.6%) were from the Philippines, seven (1.8%) were from Pakistan, four (1.1%) were from Ethiopia and Sri Lanka each, three (0.8%) were from Syria, two (0.5%) were from Saudi Arabia, while one (0.3%) patient was from Bangladesh, United States of America, Baluchistan, Egypt, Iran, United Arab Emirates, Jordan, Morocco, Uzbekistan, Yemen each and one (0.3%) patient with unknown nationality). The median of parity was 3 (IQR: 1-5), and most of the patients (n=207/333, 62.2%) were three or more parous. Two hundred and twenty-three (58.8%) patients were in the pre-menopause status while 156 (41.2%) were in the post-menopause. Family history of malignancy was positive in six (37.5%) out of the 16 (4.2%) patients with available data. The most common associated comorbidity was hypertension (n=118, 31.1%) followed by diabetes mellitus (n=103, 27.2%). Most of the patients (n=114/198, 57.6%) were obese (body mass index ≥ 30). There was no significant difference between the two groups in terms of age, nationality, smoking, parity, menopause status, history of abdominal pain, abnormal uterine bleeding, and post-menopausal bleeding, family history of malignancy, comorbidity, weight, and body mass index.

The most performed surgery was hysterectomy with or without removal of adnexa (n=314, 82.8%). The route of surgery was mainly abdominal (n=254/314, 80.9%), followed by vaginal (n=59/314, 18.8%), and one (0.32%) patient had laparoscopic-assisted vaginal hysterectomy. Symptomatic uterine leiomyoma was the most frequent indication (n=140/314, 44.6%), followed by prolapse of the uterus (n=58/314, 18.5%) (Table 2).

**Table 2:** Indications of hysterectomy with or without removal of adnexa The data has been represented as number (%).

Indications	n (%)
Symptomatic uterine leiomyoma	141 (44.9)
Prolapse uterus	58 (18.5)
Abnormal uterine bleeding (a part of symptomatic leiomyoma)	50 (15.9)
Post-menopausal bleeding	40 (12.7)
Adenomyosis	8 (2.5)
Pre-menopause with ovarian cyst	8 (2.5)
Post-menopause with ovarian cyst	6 (1.9)
Post-menopause with dermoid cyst	4 (1.3)

Sixty-five (17.2%) patients underwent myomectomy; 54 (83.1%) of them had abdominal, eight (12.3%) had vaginal, and three (4.6%) had laparoscopic myomectomy.

UM was detected in five (1.3%) patients: four (1.3%) out of 314 patients with hysterectomy and one (1.5%) out of 65 patients with myomectomy. All cases of UM were operated on abdominally. Symptomatic uterine leiomyoma was the most common surgical indication for UM (Table 3).

**Table 3:** Unexpected malignancies based on the preoperative surgical indication The data has been represented as number (%). ^a^A case of smooth muscle tumor of uncertain malignant potential and leiomyoma with bizarre nuclei was not included, and ^b^a case of mucinous borderline tumor was included. UM, unexpected malignancy; AUB, abnormal uterine bleeding.

Surgical indication	UM, n=6^a,b^	Endometrial cancer, n=1	Sarcoma, n=3	Ovarian cancer, n=2^b^
	n (%)	n (%)	n (%)	n (%)
Leiomyoma	3^a^ (50.0)	1 (100)	2 (66.7)	0 (0.0)
Ovarian cyst or adnexa mass	2 (33.3)	0 (0.0)	0 (0.0)	2 (100.0)
AUB	1 (16.7)	0 (0.0)	1 (33.3)	0 (0.0)

Six (1.6%) out of eight (2.1%) patients with unexpected malignant or premalignant pathologies had available cervical cancer screens which were all negative. Five (1.3%) of the eight (2.1%) patients had available endometrial sample histopathological reports; two (0.5%) cases showed insufficient tissue, while the other three (0.8%) cases were negative for atypia or malignancy. Tumor markers were collected for four (1.1%) patients, and they were all normal. The Risk of Malignancy Index 2 was used for patients with ovarian cysts or adnexa mass and none of the patients reached the cutoff value of 200.

Sarcoma was the most frequent UM which was detected in three (0.8%) patients (two (0.5%) were leiomyosarcoma and one (0.26%) was endometrioid stromal sarcoma), followed by squamous cell carcinoma arising from mature teratoma and endometrioid adenocarcinoma in one (0.26%) patient each. Other unexpected premalignant lesions were also detected (smooth muscle tumor of unknown malignant potential with an intravascular growth pattern, leiomyoma with bizarre nuclei, and mucinous borderline tumor of the ovary of endocervical type in one (0.26%) patient each). All UMs were at early FIGO stages (stage 1 or 2) (Table 4).

**Table 4:** Clinicopathologic details of patients with unexpected premalignant and malignant pathologies The data has been represented as number. ^a^Weight of myoma, ^b^weight of uterus, ^c^weight of ovarian cyst. yrs, years; TAH, total abdominal hysterectomy; AUB, abnormal uterine bleeding; STUMP, smooth muscle tumor of unknown potential; ESS, endometrial stromal sarcoma; EAC, endometrial adenocarcinoma; LMS, leiomyosarcoma; MBT, mucinous borderline tumor; SCC, squamous cell carcinoma; NED, no evidence of disease.

Procedure	Age (yrs)	Indication	Uterus weight (g)	Post-op histology	Additional treatment	Status (yrs)
TAH	42	Leiomyoma	350	STUMP	-	NED at 4
TAH	44	Leiomyoma	-	LM-BN	-	-
TAH	45	AUB	303	ESS low grade stage 2A	Excision of metastatic lung nodule, adjuvant radiotherapy	NED at 5
TAH	52	Leiomyoma	-	EAC grade 1 stage 1A	Adjuvant radiotherapy	NED at 3
TAH	51	Leiomyoma	900	LMS grade 3 stage 1B	Adjuvant radiotherapy	NED at 4
Myomectomy	34	Leiomyoma	300^a^, 115^b^	LMS stage 1B	Oncologic staging surgery, TAH	NED at 4
TAH	62	Ovarian cyst	395^c^, 100^b^	MBT of endocervical type stage 1A	-	NED at 4
TAH	55	Ovarian cyst	900^c^, 130^b^	SCC arising from mature cystic teratoma	-	NED at 2

For postoperative treatment of the patients with UM, three (0.8%) patients had radiotherapy and one (0.26%) patient had hysterectomy and oncologic staging surgery. The mean of follow-up was 3.7 ± 0.95 years, and none of the patients had a recurrence of malignancy or death till the end of the study. None of the studied independent variables had significantly predicted the occurrence of UM (Table 5).

**Table 5:** Results of logistic regressions for different parameters for prediction of unexpected malignancy The data has been represented as odds ratio, 95% confidence interval, log odds, or predicted probability. p-value^a^ <0.05 is considered statistically significant. OR, odds ratio; CI, confidence interval; AUB, abnormal uterine bleeding; PMB, post-menopausal bleeding; SCD, sickle cell disease; BMI, body mass index.

Variable	OR͙	95% CI	p-Value^a^	Log odds	Predicted probability (%)
Age	0.973	0.904-1.046	0.458	0.021	2.036
Nationality (n=379), Reference: Bahraini				0.026	2.500
Non-Bahraini	0.801	0.159-4.048	0.789	0.021	2.013
Smoking (n=11), Reference: non-smoker				0.500	33.333
Smoker	0.250	0.010-5.985	0.392	0.125	11.111
Parity (n=333)	0.878	0.648-1.190	0.402	0.023	2.289
Menopause status (n=380), Reference: no menopause				0.023	2.242
Menopause	0.855	0.201-3.631	0.832	0.020	1.923
Abdominal pain (n=303), Reference: no abdominal pain				0.019	1.843
Abdominal pain	2.2598	0.635-10.360	0.184	0.049	4.651
AUB (n=301), Reference: no AUB				0.020	1.935
Presence of AUB	1.797	0.422-7.656	0.428	0.035	3.425
PMB (n=233), Reference: no PMB				0.027	2.597
Presence of PMB	1.000	0.198-5.061	1.000	0.027	2.597
Family history of malignancy (n=16), Reference: no family history				0.111	10.0
Positive family history	4.50	0.310-65.229	0.270	0.500	33.333
Diabetes, Reference: nondiabetic				0.023	2.273
Diabetes	0.851	0.169-4.289	0.845	0.020	1.942
Hypertension, Reference: non-hypertensive				0.025	2.410
Hypertension	0.698	0.139-3.513	0.663	0.017	1.695
Dyslipidemia, Reference: non-dyslipidemic				0.021	2.055
Dyslipidemia	1.306	0.258-6.604	0.747	0.027	2.667
Hypothyroidism, Reference: no hypothyroidism				0.022	2.121
Hypothyroidism	0.961	0.116-7.986	0.971	0.021	2.041
Asthma, Reference: non-asthmatic				0.023	2.241
Asthmatic	0.000	0.000-0.000	0.999	0.000	0.000
SCD, Reference: non-SCD				0.023	2.204
SCD	0.000	0.000-0.000	0.998	0.000	0.000
BMI (n=198)	1.058	0.947-1.181	0.318	0.014	1.375

## Discussion

This study found that the incidence of UM among hysterectomies and myomectomies in a sample of 379 cases performed over four years was 1.3% (n=5), which is within the range reported in the literature (0-3.17%) [2,7-9]. However, the frequency of different types of UM was different; sarcoma was the most frequent cancer, followed by endometrial cancer, contrary to previously published studies which showed that endometrial cancer was the most common [1,5,11,12]. Yet, the incidence of endometrial cancer in our analysis, which was 0.26% (n=1), remained comparable to these published studies (0.26-1.02%) [1,11,12]. Although patients with endometrial hyperplasia without atypia were included in the current study, the incidence of endometrial cancer was still less than that reported by Mahnert et al. (1.02%) who also included patients with endometrial hyperplasia [12].

In the present study, there was no unexpected cervical cancer or metastatic malignancy among the cases. This finding is similar to that of Elliot et al.'s study, but contrary to other studies [5,10,12].

The median age of the patients with UM in our cohort was 48 (IQR: 43-54) years, which makes them younger than those reported in the prior studies in which the mean age ranged between 49.5 and 61 years [4,5,10,13,14]. Moreover, we reported a single patient of unexpected leiomyosarcoma whose age was less than 35 years. This emphasizes that comprehensive preoperative workup for younger patients should not be underestimated.

Our two (0.5%) patients with unexpected leiomyosarcoma had uterine weights of less than 1 kg (0.45 kg and 0.9 kg). This finding is in accordance with the existing data in the literature which showed that sarcomas exist in small uterine weights [12]. On the contrary, Multinu et al concluded that higher uterine weights were directly associated with an increase in the incidence of unexpected sarcomas. They reported four cases of leiomyosarcoma with uterine weights ranging between 1 and 2 kg [4].

In the current study, all UMs were detected among those who underwent open abdominal surgeries. This might be attributed to that the majority of our patients were operated on with open abdominal surgeries (n=308, 81.3%), while laparoscopic surgeries were limited to four (1.1%) patients. Prior study showed that UMs were more common in open abdominal surgeries [2]. However, there were reported studies in the literature which showed contrary results. For example, Ding et al. found that UMs were more common among laparoscopic-assisted vaginal hysterectomies subgroup with an incidence of 0.44% [11].

Premalignant uterine lesions in this study were the smooth uterine muscle of uncertain potential and leiomyoma with bizarre nuclei. The incidence of the first lesion was 0.26% (n=1) in a perimenopause patient who had a hysterectomy for symptomatic uterine leiomyoma. Our finding was comparable to that of Mori et al. who found this lesion in one (n=1/281, 0.36%) patient who had hysterectomy [15]. Yadav et al. reported a higher incidence (8.5%) among patients who had myomectomy, and all patients had large fibroids (>7 cm in diameter). They concluded that caution should be exercised in patients with larger-sized fibroids [2]. The probability of recurrence was between 8.7% and 11%, and it may recur as “borderline” low-grade leiomyosarcoma [16]. Our patient had a hysterectomy and thus had a favorable prognosis.

Leiomyoma with bizarre nuclei was found in another (0.26%) perimenopause patient who had a hysterectomy for symptomatic leiomyoma. Though this lesion usually runs a benign clinical course, there are a few reported cases in the literature that showed the progression of this variant to leiomyosarcoma [16]. Our patient had a good prognosis because a hysterectomy was performed.

In this study, the incidence of occult ovarian cancer was 0.26% (n=1) which is within the range of previous studies (0.19%-1.08%) [10,12]. One (0.26%) case of mucinous borderline tumor of endocervical type was also found. Both of our patients were at early FIGO stages and were not detected preoperatively, which agrees with the difficulty of recognition of borderline or early-stage ovarian cancers [2]. Risk of Malignancy Index 2 and 4 were used as a prediction model for ovarian cancer in our patients according to the recommendation of the Royal College of Obstetricians and Gynecologists (RCOG Guideline, Green Top 34, 2016) [17], but none of our cases reached the cutoff value of 200.

All patients with UM were at early FIGO stages, and this approves the described cases of occult malignancies in the literature [2,6,11,18]. Desai et al described 347 cases of uterine cancers (79.6% were at stage 1 and 7.2% were at stage 2) and 46 cases of ovarian cancers (60.9% of them were at stage 1 and 10.9% were at stage 2) [10]. Moreover, Ding et al reported 14 cases of endometrial cancer with 13 of them being at stage 1 and five cases of sarcoma which were all at stage 1 as well [11].

All our patients had their definitive treatment at a short interval postoperatively and had a favorable prognosis with no evidence of recurrence or death till the time of reporting this study. This demonstrates that the stage of the disease is an important prognostic factor of UM, in line with a prior study [13].

This study has several limitations. Being a retrospective study, our registry is expected to lack some data related to patient’s family history, smoking habits, and body mass index. Furthermore, some patients were referred cases from other hospitals. Subsequently, their radiological imaging reports, tumor markers, and preoperative histopathological specimens’ reports were not available in our institution’s electronic medical records as they were done at other institutions. In addition, there is no formal registry that collects the data of patients who underwent hysterectomy or myomectomy in our institution which makes data retrieval difficult to achieve. Moreover, the weights of postoperative specimens of patients with benign pathologies were not abstracted, and thus no comparative analysis was done between patients with benign pathology and those with UM. The small number of patients in this study may underpower its findings and limit the analysis of predictive factors of occult malignancies. Despite these limitations, this study is the first report from Bahrain, and to the best of our knowledge from the Middle East, to address occult malignancies in hysterectomies and myomectomies. All data were collected and reviewed by gynecologists, which enhances the data-gathering accuracy in comparison with the use of administrative data. We reviewed all the radiological imaging and the postoperative histopathological reports for each included case and verified the final histopathological diagnosis and the stage of the disease accurately. 

## Conclusions

Although this study demonstrated a low incidence of UM among both hysterectomies and myomectomies, the age at the diagnosis of our patients with UM was as young as 34 years, and sarcomas were the most common type of UM. Disconcertingly, none of the studied independent variables had significantly predicted the occurrence of UM. Future directions should search for predictive models and risk stratification for patients with UM, in addition to a holistic approach in triaging these lesions preoperatively to improve management strategies. 
